# The Sociology of Law potential: exploring its scientific landscape

**DOI:** 10.3389/fsoc.2023.1203799

**Published:** 2023-07-19

**Authors:** Håkan Hydén

**Affiliations:** ^1^Department of Sociology of Law, Lund University, Lund, Sweden; ^2^Academy of Learning, Humanities and Society, Halmstad University, Halmstad, Sweden

**Keywords:** norms, Norm Science, law, Sociology of Law, scientific landscape

## Introduction

The Sociology of Law (SoL) is an island of investigation squeezed between two large academic territories: the legal and the social sciences. They represent different knowledge interests based on separate ontologies, which make the epistemologies incommensurable. Law is an open normative science using interpretative, deductive methodology, while sociology has an empirical ontology built on social science methodologies in epistemological respect. The theoretical discourse to try to integrate legal dogmatic and sociology must be regarded as a dead end (Banakar, 2003; Cotterrell, 2006; Nelken, 2009, chs. 10 and 11).). The same can be said about the inside/outside dichotomy in SoL (Banakar, 2002, p. 18). SoL has a territory of its own, which is huge and to a large extent undetected and unknown. The purpose of this article is to investigate this varied landscape that is the home turf of Sociology of Law and explore the Field's contribution to knowledge. What makes it a separate entity? What is its potential?

SoL has two sides, one facing the legal science and the other facing the social sciences. It deals with law but without adding to the mainstream legal science, legal dogmatic. SoL of law uses social science theory and methods in order to study legal matters from a social and a societal perspective. This hybrid has led to a discussion about the identity of SoL (Banakar, 1998). Legal dogmatic focus on the rule of law, while Socio-legal studies have its knowledge interest on the role of law. SoL both complements and competes legal dogmatic in analyzing and understanding law and legal decision-making and it complements social science in understanding legal phenomena in their social and societal implications. From its vantage point, SoL traces a lot of gaps and cracks in the legal landscape. Filling these gaps belong to the potential for SoL. We can here talk about two different realities of law: one based on the internal operations, practices, concepts and perceptions, the other focusing on law's interaction with its societal environment (Banakar, 2001, p. 14). The first reality belongs to mainstream legal science and is in its epistemological part of no interest for SoL, while the second reality is the knowledge field for SoL, which is not relevant either for legal science.

## How SoL differs from the legal science

Sociologists of Law and legal scholars share the law as their object of study, but they approach it in different ways. While the latter concentrate on its internal operations, practices, concepts, and perceptions, the former focus on the interaction between law and its wider societal environment. Legal science has the ambition to uncover the content of law in specific cases, i.e., how the wording of legal provisions or precedents should be interpreted. The understanding of a certain phenomenon is determined deductively using legal sources. Thus, the limits for what can be discovered are set in advance. The map so to speak is already constructed. SoL, in contrast, is an open-ended field concerned foremost with the background or genesis of law and its consequences. The focus lies on the growth of law and the functions of legal regulation. SoL uses an inductive methodology aimed at relating empirical findings to theory. Defining its scientific map becomes an exploratory exercise. The more empirical findings that can be added, the richer its contours become.

### Shedding light on murky areas of the law

SoL approaches the law from an external vantage point, which means that it registers things that the lawyer involved with the dogmatics fails to see and appreciate. For example, in relation to civil law, it is interested in how contracts get used by the parties. In the case of criminal law, it is a matter of understanding the origins of the norms that define what is right and wrong. Civil and criminal law are both reactive in that they actualize things in an ex post manner (Hydén and Hydén, 2019). As rules of the game, these provisions should be as precise as possible. Administrative law is different. Its origin lies in the public sphere. It is the political system that conveys tasks to the executive about what should be carried out and provide instructions for how to go about it. As such, administrative law is goal-oriented and is applied ex ante, in advance. It contains provisions telling professional civil servants with different specialties what to do. Here is a potential for SoL research to map out how this kind of legislation is applied and with what results, a set of issues which for epistemological reasons is left untouched in legal dogmatics.

One reason for establishing SoL as a discipline relates to the need for other methods than legal dogmatic in relation to regulation about implementation and evaluation research. It was an answer to the tremendous growth of administrative and intervening law during the 1970's and onwards. Prime minister of Sweden, Olof Palme was the one who 1972 took the decision to establish SoL as an academic subject and institution. Professor Per Stjernquist who was the one introducing SoL in Sweden focused in his research on the implementation of the laws in the Forest (Stjernquist, 1973). However, the politicians do not ask for that kind of research and education. They seem not interested in research pointing out that the aims and goals of the legislation not always fulfilled.

The SoL approach to law becomes especially relevant in the case of what I call “intervening law” (Hydén, 1978, 2022), which is a mix of civil, criminal, and administrative law. This type of law is typically protective of public interests. Examples include labor laws to defend workers, environmental laws to protect nature, consumer legislation to safeguard customers, and discrimination laws to shield minorities. The source of this legislation is the conflict between different—sometimes incompatible—interests inherent in modern society. The intervening rule serves as a balancing norm; i.e., it prescribes what interests are to be protected and weighed against each other during the conflict resolution process, but it does not provide instructions for how to do so. Here are similarities to what Gunter Teubner has described in terms of reflexive law (Teubner, 1983; Rogowski, 2013). In such cases society must resort to alternative mechanisms in the political arena. Anyhow, this represent an interesting area for SoL to explore.

### Highlighting the limits of law

Legal dogmatics takes law for granted. It does not address the basic question whether the courts or public authorities really follow the law and take decisions accordingly. Law does not function in a vacuum. Its interpretation is subject to influences that may undermine a strict dogmatics approach. One example is the Swedish Compensation Act which regulates the use of public funds to pay people who are unemployed (Christensen, 1980). The result was much too inconsistent in relation to the law. Another Swedish case relates to the inpatient care of psychiatrically ill persons (Hetzler, 1978). Hetzler concludes in her study that the criteria set out in the law are rarely used. Other criteria, including social factors, weigh heavier in the final decision about these patients.

These studies follow a tradition of interest in equality before the law (Lernestedt, 2015). The Norwegian Sociologist, Vilhelm Aubert pioneered this kind of studies in the 1970's (Aubert, 1976). Comparing social status to punishment in all criminal cases in six district courts in the Eastland region of Norway in the 1950's, Aubert found significant variations between judges and courts sentencing similar cases. The mentioned kind of studies have not been followed up, but represents a wide area of potential studies within SoL. The notion that the law is not always fair is the fundamental premise of the Critical Legal Studies movement (Unger, 1986, 2015). In its perspective and that of the Scandinavian Legal Strategy movement, justice is something to fight for (Hydén, 1982; Widerberg, 1988; Bottomley and Conaghan, 1993).

## How it differs from the social sciences

The Sociology of Law is not only examining the law and its function in society. Its interest extends to the study of normative orders in other social and economic contexts (Hydén, 2022, ch. 1). As a science in which norms serve as the analytical tool, explaining motives becomes paramount. In this respect, it differs from mainstream social science where the motives behind behavior and actions are omitted. Legal science does address motive to some extent. In criminal law, for example, intent as a motive is a prerequisite for definition of most crimes. It features also in the context of administrative law. For instance, it is not possible to decide whether an object constitutes waste simply by looking at it. Whether it does, depends on the owner's intention: will he keep it or not? (Hydén, 1982, p. 135). The point here is that by focusing on the motives behind action SoL fills a gap in knowledge formation, especially in the social and behavioral sciences.

## Finding patterns in the landscape

Because norms are abstract, they are not easy to find. They can only be detected in an indirect manner through a three-pronged research process. The first step is to find and identify patterns or regularities in society. Once patterns have been identified, the second step is to determine how a specific pattern emerges.

When a certain pattern has been spotted we have—as a second step—the key to find the underlying norm, the reason why the pattern arises. The third and final step of the process addresses the why question: why do norms look like they do. This involves exploring the motives that sustain norms and carry them further. The outcome of a norm application is ultimately dependent on the possibilities of carrying out what the norm prescribes. The limits of these possibilities depend on knowledge are set by systems that humans have created to satisfy their needs.

The study of norm systems constitutes a niche that is not covered by legal and social science researchers. It contributes to knowledge formation in several ways. Firstly, norms unite cognitive and social elements, making it possible to gauge motives to act at the collective level. This is in contrast with both social psychology and sociology where studies of attitudes or opinions tend to ignore meso and macro level motives behind individual action. By combining elements of both actor theory and systems theory the voluntary Will in a science of norms is articulated and asserted by individuals and groups but is shaped and detected through learning from collective sources.

## Conclusions

The Sociology of Law with its focus on norms approaches the study of human action and behavior in ways that complement what the legal and social sciences do. By uncovering underlying motives for action through a focus on norms, SoL adds valuable insights for understanding not only how law functions in society but also how norms help sustain specific systems guided by knowledge or other criteria. In a modern society, the understanding of norms has to be extended to cover also expectations which stems from the rationality of different systems. In these cases, sanctions are not uphold either by the State apparatus or by social control. The sanctions for norm violations are embedded in the norm itself (Hydén, 2022, 7.2.1).

Technology leaves tracks of specific behavioral norms explaining by technical changes. Chamorro-Premuzic talks about humans in the AI age (Chamorro-Premuzic, 2023). He claim that we are only focused on what algorithms and artificial intelligence want us to focus on. Tracing results from personalized searches, a website algorithm selectively guesses what information a user would like to have and encapsulates the user in a filter bubble (Bozdag, 2013). The behavior impact and consequences AI has on us is huge, according to Chamorro-Premuzic.

As this article has tried to demonstrate, the scientific map that constitutes the guide for SoL research is both wide and varied. Being a young field, much remains to be investigated. One such area is the close historical connection between the stage and formation of society and the development of norms. To what extent are they the product of material forces?

For its future development, SoL has a valuable interdisciplinary potential. It responds to the present need for inter- or multidisciplinary perspectives by being synergetic. This is especially important as researchers contemplate transiting from a deconstructing, reductionist science to a constructive, holistic science. SoL provides the tool for this purpose with its scientific norm perspective based on as many points of contact with other disciplines as possible. The concept of norms in the wider sense argued for here, constitutes perhaps the most valuable tool for a synthesizing science by level the scientific playing field providing a common denominator, norms.

## Author contributions

The author confirms being the sole contributor of this work and has approved it for publication.
